# Vascular Steal Phenomenon in Lower Limb due to Reactive Hyperemia in Contralateral Limb: A Case Series and an Explanation of a Rare Phenomenon Using Continuity Equation

**DOI:** 10.3400/avd.cr.24-00019

**Published:** 2024-04-23

**Authors:** Sameer Peer, Lovleen

**Affiliations:** 1 Department of Radiodiagnosis, All India Institute of Medical Sciences, Bathinda, Punjab, India

**Keywords:** hemodynamics, blood flow velocity, angiography

## Abstract

In this series of three cases, we describe the vascular steal phenomenon in an otherwise normal lower limb secondary to hyperemia in the contralateral lower limb. In each of the cases, post-inflammatory hyperemia in the involved lower limb was associated with a significant reduction in blood flow in the contralateral normal lower limb. We attempt to explain the imaging findings in these three cases using the equation of continuity in fluid dynamics. To the best of our knowledge, a description of such kind is unavailable in the published literature.

## Introduction

Hemodynamics refers to the physical principles governing the flow of blood within the vessels in the human body. Fundamentally, blood behaves as a non-Newtonian fluid, and its viscoelastic behavior is dependent on the degree of shear stress it is subjected to.^1)^ At higher shear rates, the blood becomes less viscous and tends to follow the behavior of Newtonian fluids.^2)^ The blood flow rate in a particular region depends on various factors, such as the pulse pressure, resistance of the vascular bed, elastic properties of the conducting arteries, and the viscoelastic properties of the flowing blood.^1)^ In general, the resistance to blood flow is governed at the level of arterioles.^3)^ With the release of a myriad of vasoactive cytokines accompanying the inflammatory response, there occurs vasodilatation in the involved vascular bed, consequent to the loss of resistance to blood flow and subsequent increase in blood flow rate to that region as a reactive phenomenon, which leads to hyperemia.^4)^

Steal phenomenon refers to the rerouting of blood flow from a target vascular bed to a different vascular bed, thereby reducing the perfusion of the target vascular bed and putting it at risk for ischemia and subsequent infarction. In a reactive hyperemic state, the increased blood flow toward the inflamed region may compromise perfusion in an otherwise normal vascular bed as the blood is drawn toward the hyperemic region.

We describe a very interesting and rare observation whereby hyperemia secondary to inflammation in one lower limb leads to the drawing of blood from the contralateral, otherwise normal lower limb, at the expense of the limb perfusion, thus resulting in ischemia of the “normal” lower limb resultant upon this “steal phenomenon.” We attempt to explain this rare occurrence using the continuity equation of fluid dynamics.

## Case Reports

### Case 1

**Figure 1:**
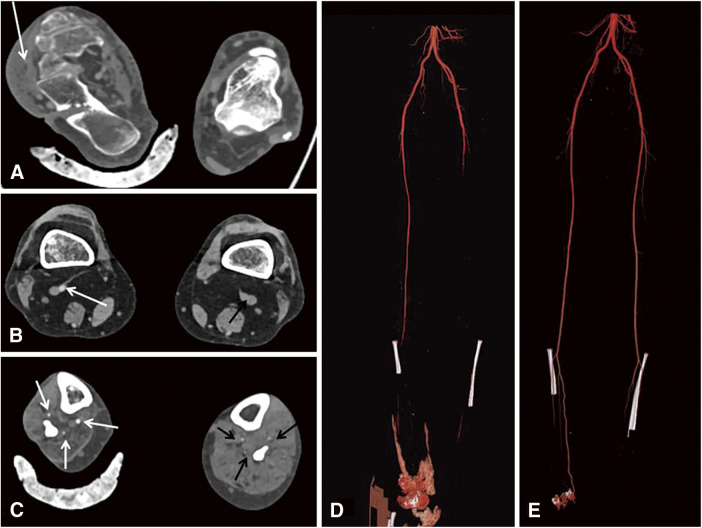
Fig. 1 (**A**) Axial CT image of the lower limbs at the level of the ankle shows diffuse subcutaneous edema involving the right lower leg and foot (white arrow). (**B**) Arterial phase CT angiography image shows luminal enhancement of the right popliteal artery (white arrow). There is no corresponding luminal enhancement of the left popliteal artery (black arrow). (**C**) The venous phase image shows good luminal enhancement of the right anterior tibial artery, posterior tibial artery, and peroneal trunk (white arrows). On the left side, faint luminal enhancement of the anterior tibial artery, posterior tibial artery, and peroneal trunk is noted (black arrows). (**D**) 3D reconstruction of CT angiography run in the arterial phase shows a considerable lag of luminal enhancement of left lower limb arteries as compared to the right side. (**E**) 3D reconstruction of the venous phase run shows further distal luminal enhancement in the left lower limb arteries, however, still lags behind the right lower limb. CT: computed tomography

A 47-year-old male patient presented with diffuse swelling of the right lower leg and foot after twisting of the right ankle. The patient was unable to bear weight on the right lower limb. No evidence of fracture was evident on X-ray examination. On clinical examination, the right foot and ankle were tender and swollen with signs of inflammation. Distal pulses in both the lower limbs were feeble. This prompted a Doppler evaluation of the lower limbs. In the Doppler study, the right lower limb arteries showed a high velocity and low resistance monophasic flow. Diffuse subcutaneous edema was noted in the right lower leg and foot. No evidence of deep vein thrombosis or arterial stenosis/occlusion was found. Interestingly, the left lower limb arteries showed low velocity and monophasic spectral waveform in the Doppler study. Computed tomography (CT) angiography of the lower limb did not reveal any steno-occlusive disease in the abdominal aorta or the lower limb arteries. On the arterial phase run, a conspicuous lag of luminal filling of the left lower limb arteries (normal side) was noted as compared to the right side (diseased side). While the right lower limb arteries up to the anterior tibial artery, peroneal artery, and posterior tibial artery were seen, the left popliteal artery did not opacify in the arterial phase run (**Fig. 1**). Subsequent venous phase scan showed further filling of the left anterior tibial artery, posterior tibial artery, and peroneal artery (**Fig 1**); however, a markedly reduced attenuation of the left lower limb arteries was seen as compared to the right side. Thus, even in the absence of steno-occlusive disease of the aorta or lower limb arteries, an attenuation of blood flow was noted in the left lower limb artery (normal side).

### Case 2

**Figure 2:**
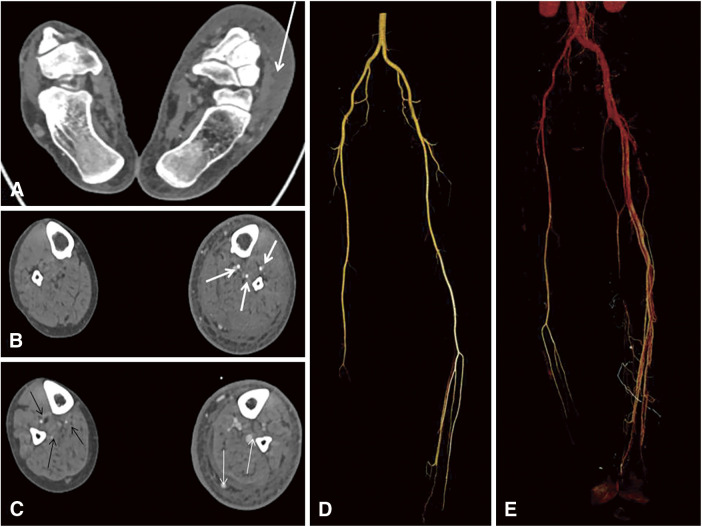
Fig. 2 (**A**) Axial CT image of the lower limbs at the level of the ankle shows diffuse subcutaneous edema involving the left lower leg and foot (white arrow). (**B**) Arterial phase CT angiography image shows luminal enhancement of the left anterior tibial, posterior tibial, and peroneal arteries (white arrows). There is no corresponding luminal enhancement of the right lower limb arteries. (**C**) The venous phase image shows good venous filling in the left lower limb (white arrows). On the right side, considerable lag is noted, with mild luminal enhancement of the right anterior tibial, posterior tibial, and peroneal arteries (black arrows). (**D**) 3D reconstruction of CT angiography run in the arterial phase shows a considerable lag of luminal enhancement of right lower limb arteries as compared to the left side. (**E**) 3D reconstruction of the venous phase run shows good venous return from the left lower limb while further distal luminal enhancement in the right lower limb arteries is noted and still lagging the left lower limb. CT: computed tomography

A 52-year-old male patient presented with complaints of edema and redness of the left lower limb for 4 days, which developed acutely. The swelling was first evident in the left foot and then progressed to involve the left leg and thigh over 4 days. The patient was a known diabetic (type II diabetes mellitus). There was no history of trauma, insect bite, or snake bite. The vitals were stable; however, a spike in fever was documented at 100^o^C. On examination, the left lower limb was obviously red, with a shiny, overlying skin that appeared tense. The left lower limb was tender and warm to the touch. While the left lower limb was obviously inflamed, the right lower limb appeared normal. However, the patient complained of occasional cramps in the right calf on moving the right foot. Doppler ultrasound examination was done primarily to rule out deep vein thrombosis in the left lower limb. While the examination ruled out deep vein thrombosis, the scan was remarkable for subcutaneous edema in the left lower limb. The left lower limb arteries showed a low flow resistance pattern with elevated peak flow velocities. Interestingly, the arterial Doppler of the right lower limb demonstrated significantly reduced peak systolic velocities and monophasic spectral waveform. No evidence of any stenosis or atheromatous plaque was noted in the iliac and femoral arteries on either side. This finding prompted CT angiography of the abdominal aorta and lower limb arteries. On a CT angiogram, the abdominal aorta and the lower limb arteries showed no evidence of any steno-occlusive disease. Hyperemia of the left lower limb was evident with thickening of the skin and subcutaneous tissues in the left lower limb, suggesting active inflammation (**Fig. 2**). Another interesting finding was the early filling of the left lower limb veins, again suggestive of hyperemia. On the right side, the arterial phase scan revealed contrast filling only up to the proximal anterior tibial artery, peroneal trunk, and posterior tibial artery. Poor distal run-off was noted in the right leg and foot. On venous phase images, the contrast column in the anterior tibial artery, posterior tibial artery, and peroneal artery was noted to have moved forward, up to the distal part of the lower leg and calf, just short of the malleolar level; however, the flow in the foot was still not evident.

### Case 3

**Figure 3:**
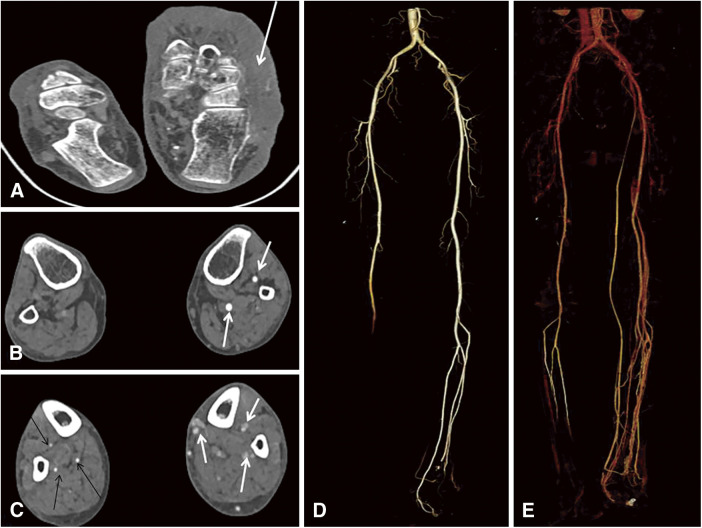
Fig. 3 (**A**) Axial CT image of the lower limbs at the level of the ankle shows diffuse subcutaneous edema involving the left lower leg and foot (white arrow). (**B**) Arterial phase CT angiography image shows luminal enhancement of the left anterior tibial and posterior tibial arteries (white arrows). There is no corresponding luminal enhancement of the right lower limb arteries. (**C**) The venous phase image shows good venous filling in the left lower limb (white arrows). On the right side, considerable lag is noted, with mild luminal enhancement of the right anterior tibial, posterior tibial, and peroneal arteries (black arrows). (**D**) 3D reconstruction of CT angiography run in the arterial phase shows a considerable lag of luminal enhancement of right lower limb arteries as compared to the left side. (**E**) 3D reconstruction of the venous phase run shows good venous return from the left lower limb while further distal luminal enhancement in the right lower limb arteries is noted and still lagging the left lower limb. CT: computed tomography

A 50-year-old man presented with complaints of swelling and redness of the left foot and leg after suffering from a scald injury to the left lower limb. There were no known comorbidities. On clinical examination, swelling of the left leg and foot and blister formation were evident. An evaluation of the distal neurovascular status revealed a very feeble pulse in the right lower limb. The distal pulses in the left lower limb were also not palpable due to diffuse edema. Thus, a Doppler examination was conducted for both lower limbs, which revealed a high velocity and monophasic flow in the left lower limb arteries. In contrast, the right lower limb arteries noted a low-velocity monophasic flow. CT angiography of the lower limbs and aorta did not reveal any steno-occlusive disease of the aorta or lower limbs. On arterial phase scan, while a prompt luminal contrast filling of the left lower limb arteries was noted, a distinct lag of contrast filling in the right lower limb arteries was evident. On the left side, the anterior tibial, posterior tibial, and peroneal arteries were seen filling, but filling only up to the popliteal artery was noted on the right side. A subsequent venous phase scan showed venous filling on the left side, while on the right side, the contrast was seen filling the anterior tibial, posterior tibial, and peroneal arteries (**Fig. 3**).

Thus, in the three cases described in this series, in the absence of steno-occlusive disease and no arterio-venous shunt, the possible explanation of the observed discrepancy of blood flow in the normal lower limb could be due to hyperemic flow in the diseased lower limb due to inflammation, with consequent “steal” of blood from the normal lower limb.

## Discussion

This series of three cases demonstrates a very rare phenomenon whereby an inflammatory response in one lower limb leads to “steal” of blood from the contralateral normal limb, resulting in ischemia of the otherwise normal limb in the absence of any steno-occlusive disease. This phenomenon could be explained by considering the continuity equation of fluid dynamics. The continuity equation applies to all fluids, irrespective of compressibility, whether the fluid is Newtonian or non-Newtonian, and can be used in explaining hemodynamic phenomenon related to blood flow in the human body.

The continuity equation is based on the basic principle of conservation of mass within a system through which the fluid flows. Consider a cylindrical structure through which fluid is flowing. If A_1_ represents the cross-sectional area of the cylinder at point 1, and A_2_ represents the cross-sectional area at point 2 along the cylinder, and if V_1_ and V_2_ refer to the velocities of blood flow at point 1 and point 2, respectively, then according to the continuity equation:

(Equation 1) $${\text{A}}_{\text{1}}{\text{V}}_{\text{1}}={\text{A}}_{\text{2}}{\text{V}}_{\text{2}}$$

Now, consider a bifurcation. Let A_1_, A_2_, and A_3_ be the cross-sectional areas and V_1,_ V_2_, and V_3_ be the blood flow rates at points A, B, and C, respectively. Then, according to the continuity equation,

(Equation 2) $${\text{A}}_{\text{1}}{\text{V}}_{\text{1}}={\text{A}}_{\text{2}}{\text{V}}_{\text{2}}+{\text{A}}_{\text{3}}{\text{V}}_{\text{3}}$$

If we consider a situation where the cross-sectional area A_3_ and blood flow rate at point C, that is, V_3_ increases, then to keep the left-hand side of the equation equal to the right-hand side, either A_2_ and/or V_2_ should decrease, or, A_1_ and/or V_1_ should increase.

The above-described illustration is analogous to the bifurcation of the abdominal aorta into two common iliac arteries. In the series of three cases that we described, the inflammatory response in one of the lower limbs resulted in vasodilatation in the arteries of that lower limb, thus increasing the cross-sectional area and velocity, considering A_3_ and V_3_, respectively, for instance. Since the mean blood flow rate at any given point in a large artery, such as the abdominal aorta, remains essentially constant at any given time, and there is negligible change in the cross-sectional area of the abdominal aorta, A_1_V_1_ may be considered constant. Thus, in a situation where A_3_V_3_ increases and A_1_V_1_ remains constant, A_2_V_2_ must decrease. Again, since the change in caliber of a large artery may be considered negligible, A_2_ may be regarded as constant. This situation leads to a reduction in V_2_, in accordance with the equation of continuity.

The above discussion explains the findings in each of the case scenarios described in this series, where inflammation in one lower limb leads to hyperemia (increase in A_3_ and V_3_, for instance), accompanied by a reduction in blood flow velocity in the contralateral lower limb arteries (reduced V_2,_ for instance). In contrast, the blood flow rate in the aorta (V_1_), the cross-sectional area of the aorta (A_1_), and the cross-sectional area of the right lower limb arteries (A_2_) may be considered constant, thus explaining the vascular steal phenomenon. In case 1, inflammation-mediated vasodilatation and hyperemia of the right lower limb resulted in reduced blood flow velocity in the left lower limb. In cases 2 and 3, inflammation and vasodilatation in the left lower limb resulted in reduced blood flow velocity in the right lower limb. We emphasize that in each of the three cases, inflammatory response in one of the lower limbs (due to trauma in case 1, infection in case 2, and scald injury in case 3) is a common clinical scenario predisposing to the occurrence of vascular steal phenomenon in the contralateral normal lower limb.

The vascular steal phenomenon has been described in arterial steno-occlusive diseases, such as the subclavian steal syndrome, whereby severe flow-limiting stenosis involving the subclavian artery, proximal to the origin of the vertebral artery results in the reversal of flow in the vertebral artery during exertion of the ipsilateral upper limb.^5)^ This occurs due to an increase in demand for blood flow in the exerting limb, which is not adequately compensated by an increase in flow from the ipsilateral subclavian artery (due to stenosis) and hence leads to “steal” of blood flow from the ipsilateral vertebral artery. A similar steal phenomenon has also been described in aorto-iliac steno-occlusive diseases and after aorto-iliac bypass surgeries, whereby mesenteric and renal hypoperfusion and/or ischemia have been described due to “steal” of blood from the mesenteric and/or renal circulation after aorto-iliac surgeries.^6)^ Some reports have also described this phenomenon post-lumbar sympathectomy.^7)^ An early description of the so-called “iliac steal” phenomenon was based on an experiment on dogs, whereby one of the iliac arteries was occluded and subsequently released.^8)^ The authors observed an increase in blood flow in the aorta and the occluded iliac artery after release of the occlusion, and a significant decrease in blood flow was also noted in the contralateral iliac artery. The findings described in the present series of three cases are fundamentally different from any of the previous descriptions of aorto-iliac steal syndrome. In this case series, we describe a steal of blood flow from one lower limb (normal limb) due to hyperperfusion-related hemodynamic changes in the contralateral limb (diseased). In these cases, no steno-occlusive disease was identified in the aorta and iliac arteries. The findings of this case series are novel, and none of the previous studies demonstrates the findings described in this series. In this series of three cases, Doppler measurements of flow velocities revealed high flow velocities in the inflamed lower limb and significantly low velocities in the normal contralateral lower limb. Also, the diameter of the arteries of the inflamed lower limb was larger than that of the contralateral lower limb. While these findings help validate our explanation of the observed vascular steal phenomenon, we propose this case series as a starting point for further research into this field of vascular hemodynamics using advanced methods of blood flow measurements in statistical measurements in a larger cohort. Also, animal studies replicating the inflammation-induced vascular steal phenomenon may be proposed based on the findings of this case series.

The teaching point and take-home message from this case series is that hemodynamic changes in one limb due to hyperperfusion can compromise blood flow in the contralateral limb, even in the absence of steno-occlusive disease, leading to the steal phenomenon. The physiological basis of this phenomenon can be explained by the equation of continuity in fluid dynamics. Vascular physicians need to be aware of this rare but interesting phenomenon and must be able to recognize the pathophysiological basis behind this phenomenon.

## Conclusion

This series of three cases describes, for the first time, the aorto-iliac steal phenomenon in the clinical scenario of hyperperfusion in one lower limb and decreased blood flow in the contralateral limb because of the steal across the aorto-iliac segment. Based on the equation of continuity in fluid dynamics, an explanation of this counterintuitive and interesting phenomenon has been provided.

## Author Contributions

Study conception: SP

Data collection: L

Analysis: SP

Investigation: L

Manuscript preparation: SP and L

Funding acquisition: Not applicable

Critical review and revision: all authors

Final approval of the article: all authors

Accountability for all aspects of the work: all authors

## Consent and Ethics Statement

The institutional ethics committee waived the need for formal approval for individual case reports and case series (order number AIIMS/IEC/2022/35). A written informed consent was obtained from the patients’ next of kin for the publication of patient-related data and images.

## Disclosure Statement

All authors declare that there are no conflicts of interest.
